# BAG3 promotes stem cell-like phenotype in breast cancer by upregulation of CXCR4 via interaction with its transcript

**DOI:** 10.1038/cddis.2017.324

**Published:** 2017-07-13

**Authors:** Bao-Qin Liu, Song Zhang, Si Li, Ming-Xin An, Chao Li, Jing Yan, Jia-Mei Wang, Hua-Qin Wang

**Affiliations:** 1 Department of Biochemistry & Molecular Biology, China Medical University, Shenyang 110001, China; 2 Key Laboratory of Cell Biology, Ministry of Public Health, and Key Laboratory of Medical Cell Biology, Ministry of Education, China Medical University, Shenyang, China

## Abstract

BAG3 is an evolutionarily conserved co-chaperone expressed at high levels and has a prosurvival role in many tumor types. The current study reported that BAG3 was induced under specific floating culture conditions that enrich breast cancer stem cell (BCSC)-like cells in spheres. Ectopic BAG3 overexpression increased CD44^+^/CD24^−^ CSC subpopulations, first-generation and second-generation mammosphere formation, indicating that BAG3 promotes CSC self-renewal and maintenance in breast cancer. We further demonstrated that mechanically, BAG3 upregulated CXCR4 expression at the post-transcriptional level. Further studies showed that BAG3 interacted with CXCR4 mRNA and promoted its expression via its coding and 3′-untranslational regions. BAG3 was also found to be positively correlated with CXCR4 expression and unfavorable prognosis in patients with breast cancer. Taken together, our data demonstrate that BAG3 promotes BCSC-like phenotype through CXCR4 via interaction with its transcript. Therefore, this study establishes BAG3 as a potential adverse prognostic factor and a therapeutic target of breast cancer.

BAG3 is a member of the human BAG co-chaperone family (BAG1–6), which interact with heat-shock protein 70 (HSP70).^1^ BAG3 has been assigned to play multiple cellular processes such as autophagy, cell survival, cytoskeleton arrangement, cellular stress response and virus replication.^2, 3^ BAG3 expression is stimulated by multiple stressful and physiological stimuli in various normal cells,^2, 4, 5, 6, 7, 8^ and inducible BAG3 expression is commonly considered as a protective mechanism upon cellular stress.^1, 9, 10, 11, 12, 13, 14^ In addition, BAG3 has been described to be upregulated and play a pro-survival role in some neoplastic tissues, including glioblastomas, pancreatic adenocarcinomas, thyroid tumors and others.^15, 16, 17, 18, 19, 20, 21, 22, 23^ However, the oncogenic potential of BAG3 remains incompletely understood.

The existence of subpopulation of cancer stem cells (CSCs) has been reported in a variety of malignancies including breast cancer.^24, 25^ A subpopulation of breast CSCs (BCSCs) existed in a growing breast tumor is supposed to contribute to radiation/chemotherapy-resistant metastasis, and function as ‘seeds’ to form new tumors after unsuccessful treatment.^24, 26^ Therefore, eradication of BCSCs is critical for breast cancer therapy, and identifying crucial molecules involved in BCSCs may provide valuable clues for therapeutic targets. BCSCs are classically defined CD44 positive and low or absent levels of CD24 expression (CD44^+^/CD24^−^^/low^), and mammosphere cultures have been used to identify BCSC-like subpopulation enriched in CD44^+^/CD24^−/low^ cells.^27, 28^

In this study, we showed that BAG3 was induced under specific floating culture conditions that enrich BCSC-like cells in spheres as compared with standard culture condition. Inducible BAG3 expression appeared to be crucial for BCSCs maintenance and renewal, as BAG3 knockdown resulted in marked decreases in first-generation and second-generation mammosphere-forming activity of breast cancer cell lines. CXCR4 is a receptor for chemokine CXCL12 and its aberrant overexpression has been implicated in BCSCs and breast tumor metastasis.^29, 30, 31, 32^ Mechanically, the current study reported that BAG3 stabilized CXCR4 mRNA via interaction with its coding region (CR) and 3′-untranslational region (3′UTR). In addition, BAG3 was found to be positively correlated with CXCR4 expression and unfavorable prognosis in patients with breast cancer. Taken together, this study establishes BAG3 as a potential adverse prognostic factor and an attractive therapeutic target for therapy directed against BCSCs.

## Results

### BAG3 is aberrantly upregulated in breast cancer and associated with poor survival

**Figure 1:**
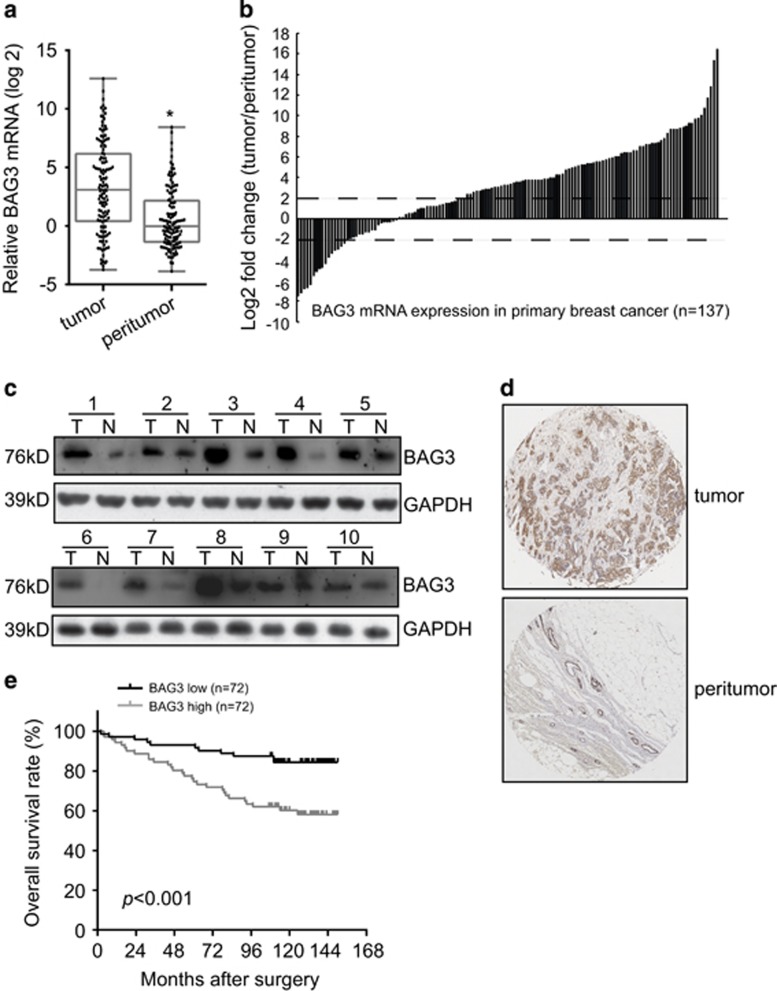
Upregulation of BAG3 expression in breast cancer correlated with poor patient survival. (**a**) BAG3 mRNA was evaluated in 137 pairs of breast cancer tissues compared with corresponding non-tumor breast specimens. BAG3 expression levels were calculated by the BAG3/18S rRNA expression ratio and plotted on the graph. (**b**) The BAG3 expression level between primary breast cancer and corresponding non-tumor breast specimens was compared. A log_2_-fold change of more than +2 or less than −2 was considered as significant upregulation or downregulation (dotted lines). (**c**) Western blot analysis of BAG3 in protein extracts from breast cancer specimens. Data are representative immunoblots of three independent assays. (**d**) Representative immunohistochemistry staining with BAG3. (**e**) Kalpan–Meier plot indicates the overall survival of patients with breast cancer categorized by BAG3 expression; *P-*value is determined by log-rank test

To investigate the potential significance of BAG3 in the progression of breast cancer, BAG3 mRNA expression was evaluated from surgical samples of 137 pairs of tumor and corresponding non-tumor breast specimens. BAG3 mRNA was significantly higher in most tumor than in peritumor breast tissues (Figures 1a and b). Immunoblot analysis of lysates obtained from surgical samples of 10 breast cancer patients confirmed increases of BAG3 expression in most tumors compared with corresponding peritumor tissues (Figure 1c). BAG3 expression was also evaluated by immunohistochemical analysis in 144 breast cancer specimens and confirmed that BAG3 expression was significantly increased in most tumor specimens relative to peritumor tissues (Figure 1d). Correlation analysis demonstrated that BAG3 intensity was positively correlated with lymphatic metastasis and estrogen receptor (ER) intensity (Supplementary Table S1). On the other hand, BAG3 intensity demonstrated no correlation with Ki67 (indicative of proliferation), progesterone receptor (PR) or HER2 (Supplementary Table S1). Survival time analysis demonstrated that patients with high BAG3 intensity showed significantly worse overall survival (Figure 1e). The Cox proportional hazards model revealed that high BAG3 was not an independent prognostic factor with respect to overall survival (hazard ratio=2.930 (95% confidence interval, 1.571–5.465), *P*=0.3413).

### BAG3 is increased during BCSC-enrichment culture and implicated in mammosphere formation of breast cancer cells

**Figure 2:**
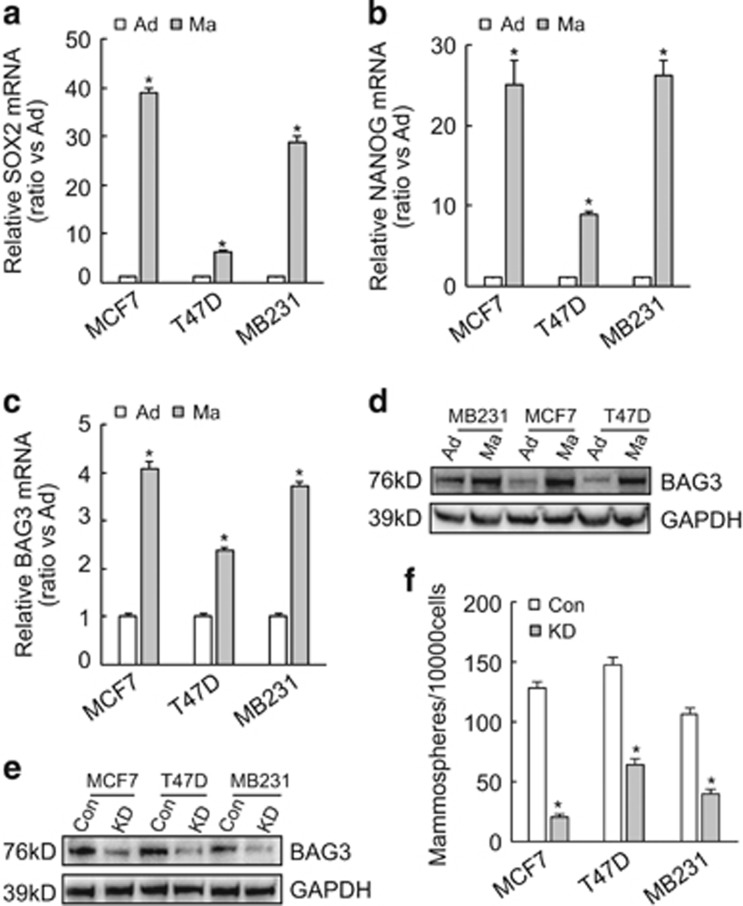
Induction of BAG3 upon BCSC-enrichment culture. (**a** and **b**) Breast cancer cells were cultured under traditional adhesive (Ad) or mammosphere-forming (Ma) culture, cancer stem cell markers SOX2 (**a**) and NANOG (**b**) expression was analyzed by real-time PCR. (**c** and **d**) Breast cancer cells were cultured as above, BAG3 mRNA (**c**) and protein (**d**) expression was analyzed by real-time PCR and western blot, respectively. (**e**) Breast cancer cells were infected with lentivirus containing empty (Con) or gRNA directed BAG3 (KD); BAG3 expression was analyzed by western blot. (**f**) Control (Con) or BAG3 knockdown (KD) breast cancer cells were cultured under mammosphere-forming condition. Mammospheres were photographed by phase-contrast microscopy and the number of mammospheres was counted, and was represented as the mean±S.E.M. from three independent experiments. NS, not significant; *P<0.01

Expression of stemness-related genes including SOX2 (Figure 2a) and NANOG (Figure 2b) confirmed that mammosphere-forming culture of breast cancer cells enriched BCSC-like cells. Importantly, real-time PCR (Figure 2c) and western blot (Figure 2d) demonstrated that BAG3 expression was also increased during floating culture of breast cancer cells compared with their adhesive partners. To explore the potential role of BAG3, BAG3 was knockdown using the CRISPR/Cas9 system in breast cancer cells (Figure 2e). The number of mammospheres significantly decreased in breast cancer cells with BAG3 knockdown (Figure 2f).

### Ectopic BAG3 expression promotes self-renewal of BCSC-like cells *in vitro*

**Figure 3:**
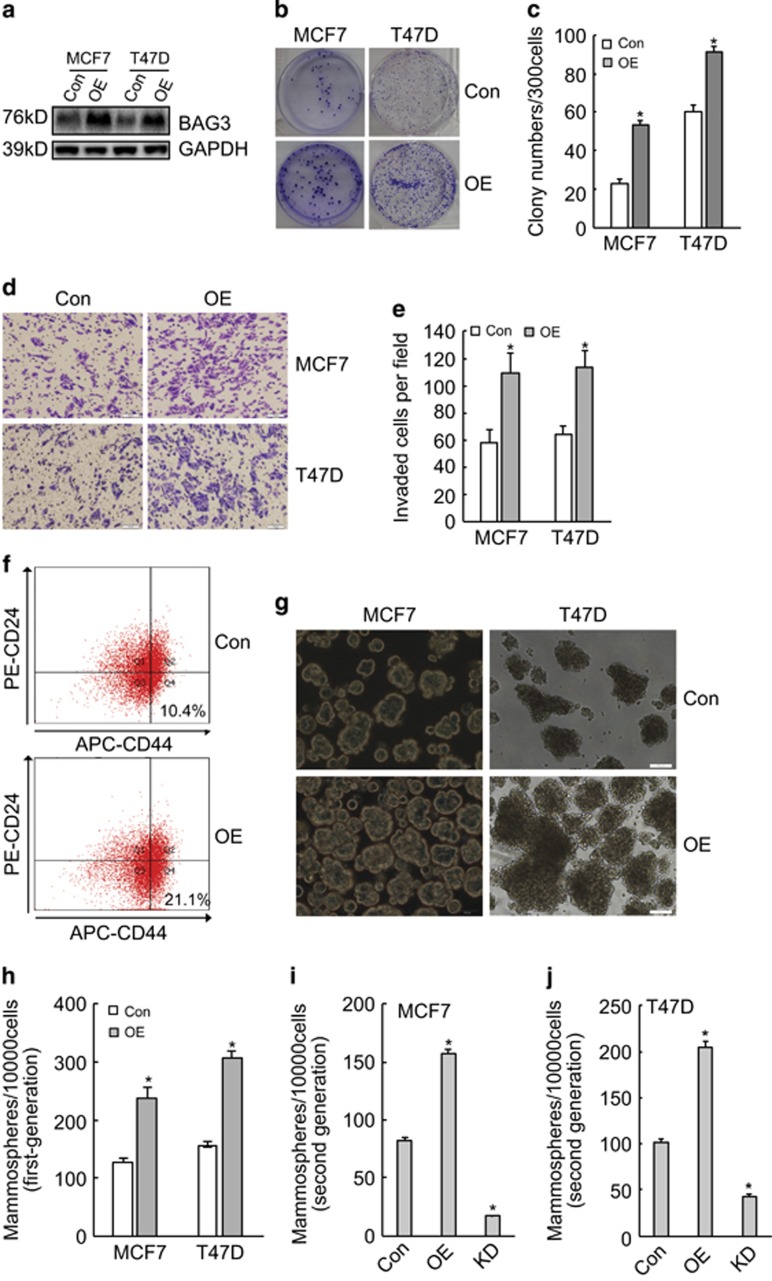
Ectopic BAG3 promoted BCSC-like properties *in vitro*. (**a**) MCF7 and T47D cells were transduced with lentivirus containing empty (Con) or BAG3 construct (OE). BAG3 expression was analyzed using western blot. (**b** and **c**) Three hundred cells were plated on a six-well plate and cultured for 14 days. Representative photographs of plate colony formation were provided (**b**), and the number of colony was counted and represented as the mean±S.E.M. from three independent experiments (**c**). (**d** and **e**) The invasiveness of control or BAG3-overexpressing MCF7 and T47D cells was evaluated by a Matrigel-coated Transwell assay. Cells that passed through Matrigel for 24 h were stained with crystal violet. Representative photographs were provided (**d**), and cells were counted and represented as the mean±S.E.M. from three independent experiments (**e**). (**f**) The subpopulation of CD44^+^/CD24^−/low^ cells from MCF7 cells was measured by flow cytometry. (**g**) Con or OE breast cancer cells were cultured under mammosphere-forming condition. Mammospheres were photographed by phase-contrast microscopy and representative images were provided. (**h**) The number of mammospheres was counted, and was represented as the mean±S.E.M. from three independent experiments. (**i** and **j**) Control, BAG3-overexpressng (OE) or BAG3 knockdown (KD) MCF7 (**i**) or T47D (**j**) cells were cultured under mammosphere-forming condition for 7 days; the first-generation mammospheres were disaggregated and single-cell suspensions were cultured under the traditional condition for 3 days, and then followed by floating culture. Mammospheres were photographed and the number of mammospheres was counted. NS, not significant; *P<0.01

To further investigate the significance of BAG3 induction during mammosphere-forming culture, BAG3 was ectopically expressed in breast cancer cells (Figure 3a). The clonogenic potential of breast cancer cells was analyzed in limited dilution assays, which revealed that ectopic BAG3 expression increased clonogenicity compared with control cells (Figures 3b and c). Transwell invasion experiments demonstrated that ectopic BAG3 expression promoted invasion of breast cancer cells (Figures 3d and e). BAG3 significantly promoted BCSC-like properties as evidenced by increase in the fraction of CD44^+^/CD24^−^ subpopulation (Figure 3f), as well as a significant increase in the number and size of mammospheres derived from breast cancer cells (Figures 3g and h). In addition, ectopic BAG3 overexpression increased second-generation mammosphere frequency in both MCF7 (Figure 3i) and T47D (Figure 3j) cells. On the contrary, BAG3 knockdown resulted in significant decrease in second-generation mammosphere frequency (Figures 3i and j). These results indicated that BAG3 affect self-renewal capacity of breast cancer cell lines *in vitro*.

### BAG3 overexpression increases mammosphere formation capacity of breast cancer cells via upregulation of CXCR4

**Figure 4:**
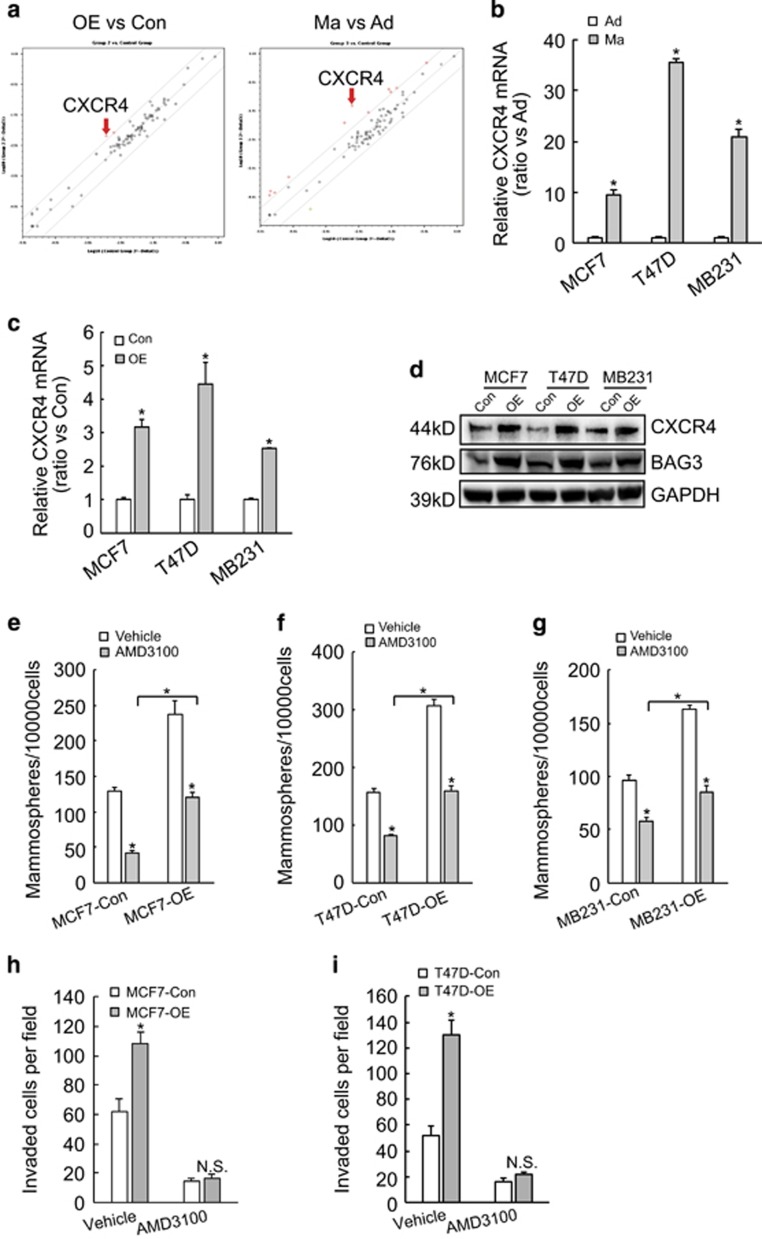
BAG3 promotes mammosphere-forming capacity of breast cancer cells via upregulation of CXCR4**.** (**a**) RT^2^ profiler PCR array was performed using total RNA isolated from control (Con) or BAG3-overexpressing (OE) MCF7 cells, traditional adhesive (Ad) or mammosphere-forming (Ma) cultured MCF7 cells, and CXCR4 was identified as one of significantly increased molecule in both OE *versus* Con and Ma *versus* Ad. (**b**) Breast cancer cells were cultured under traditional or mammosphere-forming condition, and real-time PCR was performed to measure CXCR4 mRNA expression. (**c** and **d**) Breast cancer cells transduced with empty of BAG3 construct were cultured under traditional condition, and CXCR4 mRNA (**c**) and protein (**d**) expression was analyzed using real-time PCR and western blot analysis, respectively. (**e–g**) Control or BAG3-overexpressing MCF7 (**e**), T47D (**f**) and MDA-MB-231 (**g**) cells were cultured under mammosphere-forming condition in the presence of vehicle or AMD3100 for 7 days and the number of mammospheres was counted. (**h** and **i**) The invasiveness of control or BAG3-overexpressing MCF7 (**h**) or T47D (**i**) in the presence of vehicle or AMD3100 was evaluated by a Matrigel-coated Transwell assay. Cells that passed through Matrigel were counted and represented as the mean±S.E.M. from three independent experiments. NS, not significant; *P<0.01

To explore the possible mechanisms implicated in promotion of mammosphere formation by BAG3, PCR array was performed using total RNA isolated from MCF7 cells and found that both ectopic BAG3 overexpression and BCSC-enrichment culture increased CXCR4 mRNA compared with their control partners (Figure 4a). Real-time PCR confirmed that floating culture (Figure 4b) and BAG3 overexpression (Figure 4c) increased CXCR4 mRNA levels in breast cancer cells. Consistent with mRNA expression, western blot demonstrated that forced BAG3 expression increased CXCR4 protein levels in breast cancer cells (Figure 4d). To explore the significance of CXCR4, AMD3100 was used to antagonize the function of CXCR4. BCSC-enrichment culture demonstrated that AMD3100 significantly suppressed mammosphere-forming capacity of breast cancer cells in both control and BAG3 ectopically expressed cells (Figures 4e–g). Even in the presence of AMD3100, breast cancer cells with BAG3 expression had higher mammosphere-forming activity compared with their control partners (Figures 4e–g). AMD3100 also significantly decreased invasiveness of both control and ectopically BAG3 expressed MCF7 (Figure 4h) or T47D (Figure 4i) cells. In the presence of AMD3100, cells with ectopic BAG3 expression had similar invasive capacity with their control partners (Figures 4h and i).

### BAG3 increases the half-time of CXCR4 mRNA

**Figure 5:**
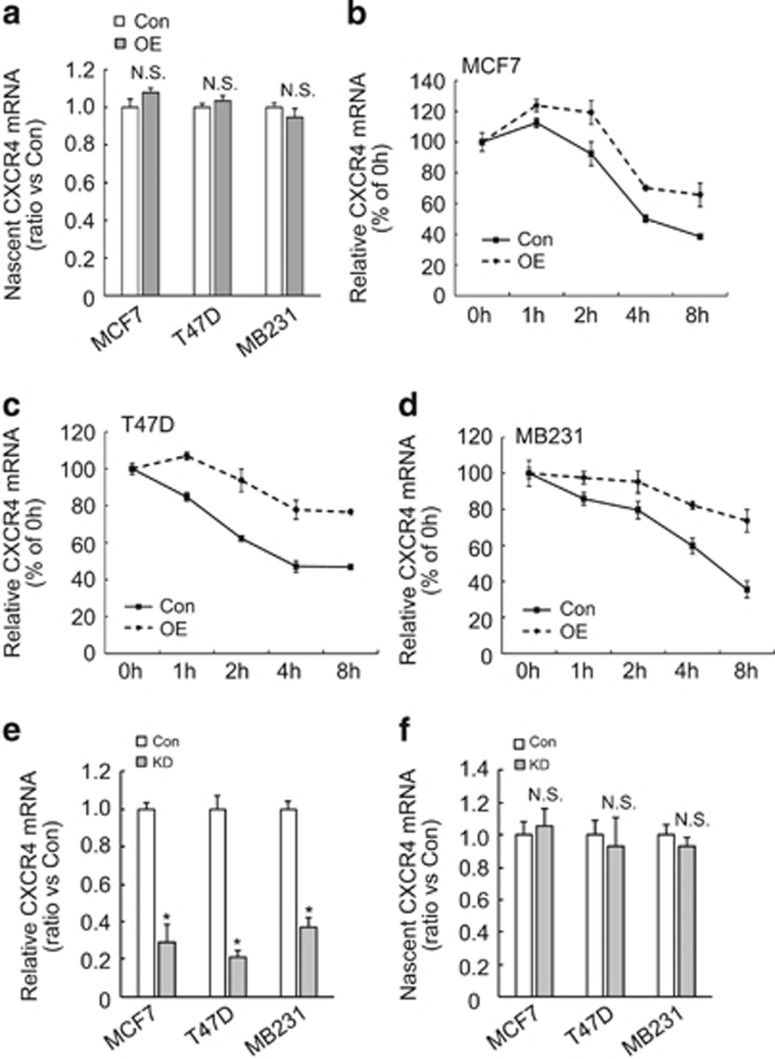
BAG3 increases stability of CXCR4 mRNA in breast cancer cells. (**a**) Nascent RNA was labeled and isolated; newly synthesized CXCR4 mRNA was analyzed using real-time RT-PCR in control or BAG3-overexpressing cells. (**b–d**) Actinomycin D was added for the indicated period to block RNA synthesis, and CXCR4 mRNA was analyzed using real-time RT-PCR in control or BAG3-overexpressing MCF7 (**b**), T47D (**c**) or MD-MBA-231 (**d**) cells. (**e**) Total RNA was isolated from control or BAG3 knockdown cells, and CXCR4 mRNA was analyzed using real-time PCR. (**f**) Nascent RNA was labeled and isolated; newly synthesized CXCR4 mRNA was analyzed using real-time RT-PCR in control or BAG3 knockdown cells. NS, not significant; *P<0.01

To further address the mechanism underlying upregulation of CXCR4 by BAG3, nascent RNA was isolated and real-time RT-PCR demonstrated that BAG3 overexpression did not alter nascent CXCR4 mRNA in breast cancer cells (Figure 5a), indicating that BAG3 promoted CXCR4 expression independent of transcription initiation. We further performed mRNA half-life experiments by blocking transcription with actinomycin D in the presence or absence of BAG3. The half-life of CXCR4 mRNA in MCF7 cells with ectopic BAG3 overexpression was markedly longer than that observed in control vector-transfected cells (Figure 5b). Similar findings were obtained in T47D (Figure 5c) and MDA-MB231 cells (Figure 5d). Real-time RT-PCR demonstrated that BAG3 knockdown decreased total CXCR4 mRNA (Figure 5e), while nascent CXCR4 mRNA was unaltered (Figure 5f) in breast cancer cells. These data indicated that BAG3 stabilized CXCR4 mRNA in breast cancer cells.

### BAG3 interacts with and stabilizes CXCR4 mRNA via its coding region and 3′-untranslational region

**Figure 6:**
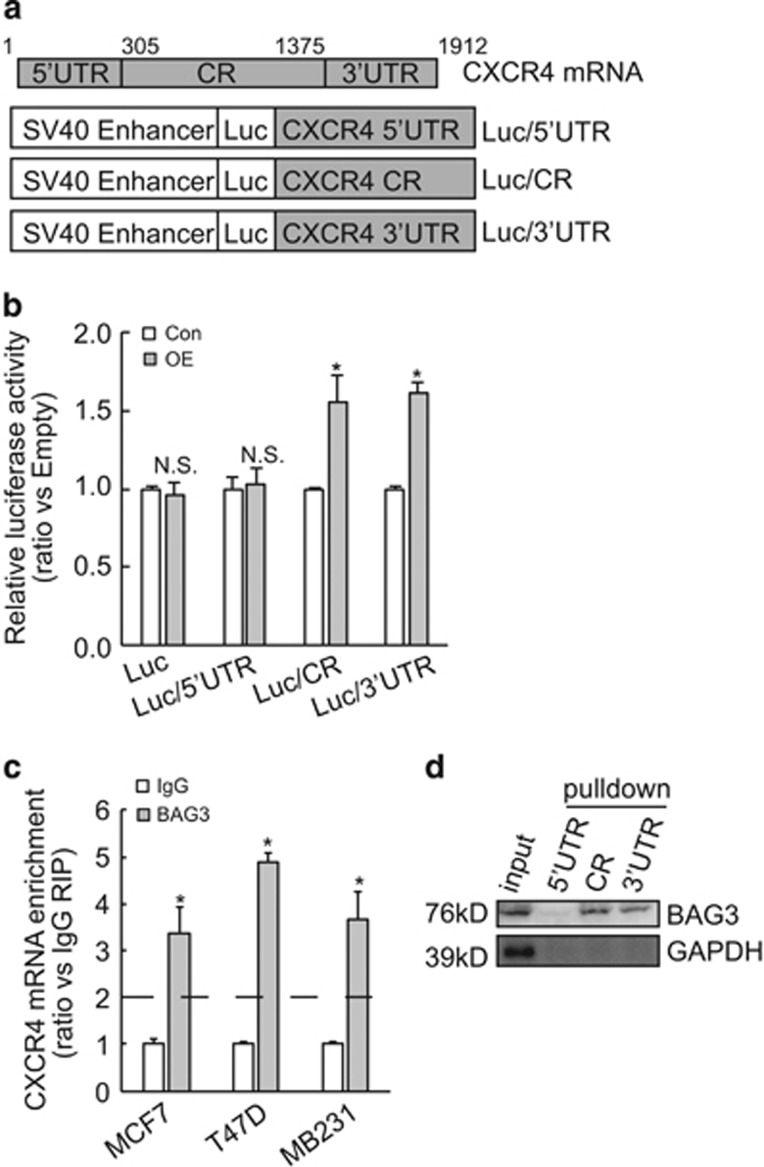
BAG3 interacts with CR and 3′UTR of CXCR4 mRNA and increases its lifetime. (**a**) Schematic representation of the luciferase reporter vector bearing 5′-untranslational region (UTR), coding region (CR) or 3′UTR. (**b**) Control or BAG3-overexpressing MCF4 cells were transfected with the indicated luciferase reporter vector and a Renilla reporter vector. Luciferase activity was measured 2 days after transfection and Renilla activity was measured to normalize luciferase activity. (**c**) RIP was performed using BAG3 antibody with lysates from breast cancer cells. CXCR4 mRNA enrichment was analyzed using real-time RT-PCR. (**d**) Biotinylated RNA segment of CXCR4 mRNA was used to pulldown lysates from MCF7 cells, and the pulldown materials were analyzed by western blotting analysis. NS, not significant; *P<0.01

To screen which segment of CXCR4 mRNA is responsible for its stabilization mediated by BAG3, 5′-untranslational region (5′UTR), coding region (CR), and 3′UTR of CXCR4 fragments were inserted to the 3′-terminus of luciferase gene (Figure 6a). Luciferase activity assay demonstrated that BAG3 significantly increased luciferase activity of reporter construct containing CR or 3′UTR fragment of CXCR4, while had no effect on control or construct containing 5′UTR of CXCR4 (Figure 6b). Very recently, our data indicated that BAG3 regulated expression of some genes at the post-transcriptional level via interaction with their transcripts (unpublished data). We then explored whether BAG3 stabilized CXCR4 transcript via interaction. RNA immunoprecipitation (RIP) demonstrated that BAG3 was recruited to CXCR4 mRNA in breast cancer cells (Figure 6c). Biotin pulldown was then performed using *in vitro* transcribed 5′UTR, CR or 3′UTR segment of CXCR4 mRNA. Consistent with luciferase analysis (Figure 6b), BAG3 was precipitated by CR and 3′UTR segments of CXCR4 mRNA, while 5′UTR segment was unable to pulldown BAG3 (Figure 6d).

### Clinical correlation of BAG3 with CXCR4 in breast cancer specimens

**Figure 7:**
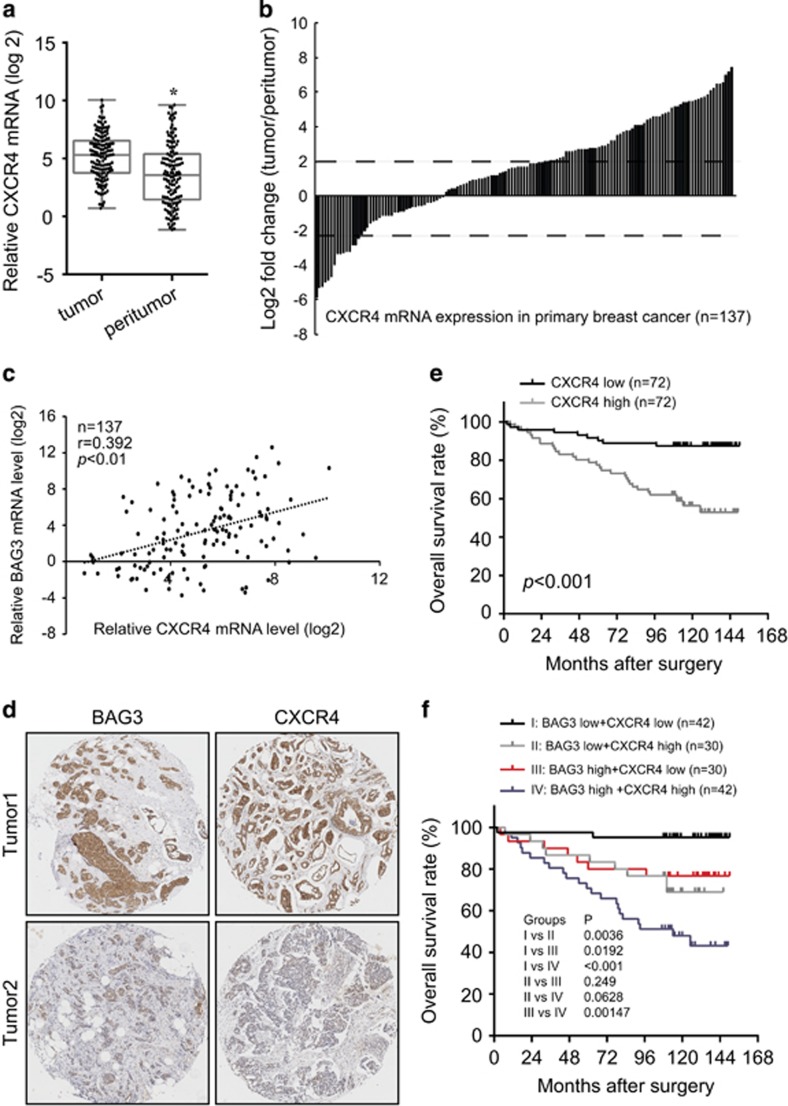
Positive correlation of BAG3 with CXCR4 in breast cancer**.** (**a**) CXCR4 mRNA was evaluated in 137 pairs of breast cancer tissues compared with corresponding non-tumor breast specimens. CXCR4 expression levels were calculated by the CXCR4/18S rRNA expression ratio and plotted on the graph. (**b**) The CXCR4 expression level between primary breast cancer and corresponding non-tumor breast specimens was compared. A log_2_-fold change of more than +2 or less than −2 was considered as significant upregulation or downregulation (dotted lines). (**c**) Regression analysis was performed between the normalized BAG3 and CXCR4 mRNA. Each dot represents a sample, and the dotted line represents the linear regression fit, with the Pearson correlation coefficient (*r*) shown in the corner of the box. (**d**) Representative immunohistochemistry staining with BAG3 and CXCR4. (**e**) Kalpan–Meier plot indicates the overall survival of patients with breast cancer categorized by CXCR4 expression; *P*-value is determined by log-rank test. (**f**) Kalpan–Meier plot indicates the overall survival of patients with breast cancer categorized by combined BAG3 and CXCR4 expression; *P-*value is determined by log-rank test

Given that BAG3 might regulate CXCR4 in breast cancer, real-time PCR was performed to evaluate the relationship between BAG3 and CXCR4. Similar like BAG3, CXCR4 mRNA was significantly upregulated in breast cancer tumor specimens compared with corresponding non-tumor tissues (Figures 7a and b). CXCR4 mRNA level was positively correlated with BAG3 mRNA expression in breast tumor tissues (Figure 7c). Immunohistochemical analysis confirmed that BAG3 and CXCR4 intensities were positively correlated in most breast cancer specimens (Figure 7d). Correlation analysis demonstrated that CXCR4 intensity was positively correlated with lymphatic metastasis, Ki67 and HER2 intensities (Supplementary Table S2). On the other hand, BAG3 intensity demonstrated no correlation with ER or PR intensity (Supplementary Table S2). Analysis of patient outcome demonstrated that high CXCR4 expression predicted poor overall survival of patients with breast cancer (Figure 7e). The Cox proportional hazards model revealed that high CXCR4 was not an independent prognostic factor with respect to overall survival (hazard ratio=3.737 (95% confidence interval, 1.998–6.989), *P*=0.2676). Considering positive correlation between BAG3 and CXCR4 expression, their combined influence on patient outcome was evaluated. BAG3 and CXCR4 expression pattern was significantly among different molecular subtypes (Supplementary Table S3). Patients with BAG3 high/CXCR4 high showed worse overall survival than those with BAG3 high/CXCR4 low (Figure 7f), confirming the involvement of CXCR4 in oncogenic function of BAG3 in breast cancer. On the other hand, patients with BAG3 high/CXCR4 low showed significantly worse overall survival than those with BAG3 low/CXCR4 low (Figure 7f), suggesting that BAG3 might possess CXCR4-independent function. Patients with BAG3 low/CXCR4 high demonstrated worse overall survival compared with BAG3 low/CXCR4 low, as well as patients with BAG3 low/CXCR4 high and those with BAG3 high/CXCR4 high demonstrated similar overall survival (Figure 7f), indicating that CXCR4 also exerted oncogenic function independent of BAG3 in breast cancer.

## Discussion

BAG3 is a pro-survival co-chaperone that is highly expressed in various tumors and its aberrant upregulation is correlated with the poor prognosis of some cancers, including pancreatic, glioblastoma and thyroid.^15, 16, 17, 18, 19, 20, 21, 22, 23^ Consistent with these reports, the current study reported that BAG3 was aberrantly upregulated in breast cancer tissues, and a high BAG3 expression level predicted poor overall survival of patients with breast cancer.

Many studies have reported that mammosphere formation *in vitro* is associated with CSCs, primarily using cultured cell lines. The current study observed that BAG3 expression is significantly induced under mammosphere-forming culture conditions as compared with traditional monolayer culture condition. Importantly, we observed a positive correlation between mammosphere-forming capacity and BAG3 expression in breast cancer cell lines. In addition, positive correlation between BAG3 expression and second-generation mammosphere formation indicated a role of BAG3 in maintenance and self-renewal of BCSC-like subpopulation. During preparing the manuscript, Im *et al.*^33^ demonstrated that BAG3 was increased during glioblastoma stem cells-enrichment culture. Consistent with our study, BAG3 appeared to confer GCSCs-like properties.^33^

As an essential molecular mechanism underlying regulation of BCSCs properties, this study identified CXCR4 as a novel target of BAG3 and a crucial mediator of BAG3’s role in promoting BCSC-like properties. CXCR4 is the most common chemokine receptor detected in the stem cell population of some tumors including breast cancer.^34, 35^ Our study demonstrated that BAG3 stabilized CXCR4 transcript at the post-transcriptional level via interaction with its CR and 3′UTR. Post-transcriptional mechanisms have attracted increasing attention since discovery of noncoding RNAs, which confer cells to alter their gene expression program rapid than transcriptional mechanisms. Besides noncoding RNAs, RNA-binding proteins (RBPs) also regulate multiple post-transcriptional processes via recruitment to their cognate RNAs.^36^ Recruitment of proteins to their cognate RNAs forms ribonucleoprotein particles (RNPs), which participate in every aspect of RNA life, from transcription, to transport, storage and translation.^36^ Throughout their life from biosynthesis to degradation, the interplay between mRNA and a variable set of proteins determines the fate of an mRNA.^36^ The current study identified interaction of BAG3 with CXCR4 mRNA using two reciprocal methods RIP and biotin pulldown. Different from miRNAs, which regulate target mRNAs via their 3′UTR, RBPs regulate their cognate mRNAs throughout the entire transcript, including 5′UTR, CR and 3′UTR.^37^ The current study demonstrated that BAG3 interacted with both CR and 3′UTR segment of CXCR4 mRNA. In addition, BAG3 increased luciferase activity of construct containing both CR and 3′UTR. These data indicated that BAG3 upregulated CXCR4 via interaction with its mRNA.

Taken together, this is the first study to describe a novel role of BAG3 in post-transcriptional regulation of CXCR4 via interaction with its transcript in breast cancer, by which promotes BCSC-like properties. Our results suggest that targeting BAG3 may result in further treatment avenues for therapy directed against BCSCs.

## Materials and Methods

### Knockout of BAG3 by CRISPR/Cas9

A dual gRNA approach was used to knock out BAG3 by the CRISPR/Cas9 system. To facilitate the selection of positive clones, a donor vector was generated in such a way that targeting sequence is replaced by marker genes (GFP and PU, the puromycin-resistance gene) once it is integrated into the genomic DNA by homologous recombination. The dual gRNA construct carrying Cas9 and donor vector were introduced into breast cancer cells by infection. The empty dual gRNA vector served as a control. One week later, the infected cells were subject to puromycin selection. Initial identification of knockout was carried out by genomic PCR, followed by real-time RT-PCR and western blot.

### Mammosphere formation assay

Cells were plated in six-well plated for 24 h and treated with AMD3100 (100 nM). After incubation for 72 h, cells were harvested and plated at a density of 3 × 10^4^ cells/well in ultra-low-attachment six-well plates (Corning, Acton, MA, USA) in serum-free DMEM/F12 supplemented with B27 (1:50, Invitrogen, Carlsbad, CA, USA), 20 ng/ml human recombinant epidermal growth factor (Sigma-Aldrich, Saint Louis, MO, USA), 20 ng/ml basic fibroblast growth factor (Sigma-Aldrich), 4 *μ*g/ml heparin (Sigma-Aldrich) and 5 *μ*g/ml insulin (Sigma-Aldrich). Mammospheres were imaged and counted under phase-contrast microscopy after 7 days of cell seeding. Only the mammospheres exceeding 50 *μ*m in diameter were counted. For the second-generation mammosphere formation, the first-generation mammospheres were disaggregated and single-cell suspensions were cultured under the traditional condition for 3 days, and then followed by floating culture. The second-generation mammospheres were scored after 7 days of cell plating.

### Colony formation assay

For the plate colony formation assay, 300 cells/well were seeded into the six-well plates and routinely cultured for 2 weeks. The cells were then fixed with 4% paraformaldehyde for 15 min and stained with 0.1% crystal violet. The colony (containing more than 50 cells) number was determined under an optical microscopy.

### Biotin pulldown assay

cDNA was used as a template for the PCR amplification of the different fragments of CXCR4 mRNA. All 5′ primers contained the T7 promoter sequence CCAAGCTTCTAATACGACTCACTATAGGGAG-3′(T7). For biotin pulldown assays, PCR-amplified DNA was used as the template to transcribe biotinylated RNA by using T7 RNA polymerase in the presence of biotin-UTP. RNA-protein binding reactions were performed using 500 *μ*g of cell lysates and 1 *μ*g biotin-labeled RNA in a final volume of 20 *μ*l using Binding Buffer A (20 mM HEPES-KOH at pH 7.5, 2.5 mM magnesium chloride (MgCl_2_), 100 mM KCl, 20% glycerol, 0.5 mM dithiotheritol and protease inhibitor tablets). Reaction mixtures were incubated for 1 h at room temperature. Complexes were isolated with paramagnetic streptavidin-conjugated Dynabeads (Invitrogen), and the pulldown materials were analyzed by western blotting analysis.

### RNA immunoprecipitation

Magna RIP RNA-binding protein immunoprecipitation kit (Millipore, Billerica, MA, USA) was used for RIP procedures according to the manufacturer’s protocol. BAG3 antibody was used to pull down CXCR4 mRNA. After the antibody was recovered by protein A/G beads, standard real-time RT-PCR was performed to detect CXCR4 mRNA in the precipitates.

### mRNA half-life measurement

To measure the half-life of endogenous CXCR4 mRNA, the expression of CXCR4 mRNA was shut off by adding actinomycin D (5 *μ*g/ml) into the cell culture medium, and total RNA was prepared at the times indicated and subjected to real-time RT-PCR analysis using CXCR4-specific primers. Data were plotted as the means±standard deviations (S.D.) from three independent experiments.

### Transient transfection and luciferase reporter assay

Cells were transfected with each plasmid construct plus plasmids carrying Renilla luciferase as an internal control. The transfection was carried out with Lipofectamine 2000 (Invitrogen) according to the manufacturer’s instructions. Cells were incubated for 24 h and harvested by adding 100 *μ*l of reporter lysis buffer (Luciferase Assay System; Promega, Madison, WI, USA). The activity of luciferase was measured using a luminometer (Männedorf, Switzerland). Firefly luminescence was normalized to Renilla luminescence. Results are presented relative to normalized luminescence driven from pGL3-promoter and reported as relative luciferase units (RLU). All experiments were done in triplicate and independently performed at least three times.

### Label and Capture Nascent RNA

Newly synthesized RNA was labeled and isolated using Click-iT Nascent RNA Capture Kit (Invitrogen) as previously reported.^38^ Briefly, nascent RNAs were labeled with 0.2 mM of 5-ethynyl uridine (EU), followed by biotinylation and isolation using streptavidin magnetic beads.

### Western blot analysis

Total cellular proteins were extracted using lysis buffer containing 20 mM Tris-HCl, 150 mM NaCl, 2 mM EDTA, 1% Triton-X100 and protease inhibitor cocktail (Sigma-Aldrich). Extracted proteins were quantified using the BCA protein assay kit. Twenty micrograms of total proteins were separated using 12% SDS-PAGE and transferred to PVDF membrane (Millipore Corporation).

### Tissue microarray and immunohistochemical staining

Tissue microarray sections were purchased from Shanghai Outdo Biotech Co., Ltd. Tissue sections was immunostained with antibodies to BAG3 and CXCR4. A semiquantitative H-score ranged was calculated for each specimen by multiplying the distribution areas (0–100%) at each staining intensity level by the intensities (0: negative; 1: weak staining; 2: moderate staining; 3: strong staining) as previously reported.^39^ The median value of the *H*-score was chosen as the cutoff criterion to dichotomize into high and low expression subgroup.

### Statistics

The statistical significance of the difference was analyzed by ANOVA and *post hoc* Dunnett’s test. Statistical significance was defined as *P*<0.05. All experiments were repeated three times, and data were expressed as the mean±S.D. (standard deviation) from a representative experiment.
