# Supplementary material for: BAG3 promotes stem cell-like phenotype in breast cancer by upregulation of CXCR4 via interaction with its transcript
Source: Cell Death Dis. 2017 Jul 13;8(7):e2933–. doi: 10.1038/cddis.2017.324 (PMC5550869; doi:10.1038/cddis.2017.324)
Supplement: Supplementary Table 1 [file cddis2017324x1.docx]

|  | BAG3 intensity | |
| --- | --- | --- |
|  | Pearson correlation coefficient  (R) | statistical significance  (*P*) |
| lymphatic metastasis | 0.22 | 0.008* |
| Ki67 intensity | -0.115 | 0.171 |
| ER intensity | 0.519 | <0.001* |
| PR intensity | 0.043 | 0.608 |
| HER2 intensity | -0.101 | 0.226 |

Supplementary Table 1 Correlation analysis of BAG3 with pathological features of breast cancer

* Significant difference in statistics
