# Supplementary material for: BAG3 promotes stem cell-like phenotype in breast cancer by upregulation of CXCR4 via interaction with its transcript
Source: Cell Death Dis. 2017 Jul 13;8(7):e2933–. doi: 10.1038/cddis.2017.324 (PMC5550869; doi:10.1038/cddis.2017.324)
Supplement: Supplementary Table 2 [file cddis2017324x2.docx]

|  | CXCR4 intensity | |
| --- | --- | --- |
|  | Pearson correlation coefficient  (R) | statistical significance  (*P*) |
| lymphatic metastasis | 0.227 | 0.006* |
| Ki67 intensity | 0.182 | 0.029* |
| ER intensity | -0.039 | 0.639 |
| PR intensity | -0.111 | 0.184 |
| HER2 intensity | 0.760 | <0.001* |

Supplementary Table 2 Correlation analysis of CXCR4 with pathological features of breast cancer

* Significant difference in statistics
