# Supplementary material for: BAG3 promotes stem cell-like phenotype in breast cancer by upregulation of CXCR4 via interaction with its transcript
Source: Cell Death Dis. 2017 Jul 13;8(7):e2933–. doi: 10.1038/cddis.2017.324 (PMC5550869; doi:10.1038/cddis.2017.324)
Supplement: Supplementary Table 3 [file cddis2017324x3.docx]

Supplementary Table3 BAG3/CXCR4 expression and molecular subtype

| Molecular subtype  ER\PR\HER | n | High/  High | High  /Low | Low /High | Low  /Low | *P* |
| --- | --- | --- | --- | --- | --- | --- |
| Luminal A | 61 | 18 | 20 | 6 | 17 | 0.00094^*^ |
| Luminal B(HER2 negative) | 12 | 4 | 1 | 2 | 5 |  |
| Luminal B(HER2 positive) | 21 | 8 | 2 | 9 | 2 |  |
| HER2 positive | 20 | 4 | 1 | 9 | 6 |  |
| Triple negative | 30 | 10 | 4 | 3 | 13 |  |

* Significant difference in statistics
